# Smart Nucleic Acid Hydrogel-Based Biosensors: From Molecular Recognition and Responsive Mechanisms to Applications

**DOI:** 10.3390/bios15120799

**Published:** 2025-12-05

**Authors:** Lu Xu, Longjiao Zhu, Xiaoyu Wang, Wenqiang Zhang, Xiaoyun He, Yangzi Zhang, Wentao Xu

**Affiliations:** 1 Food Laboratory of Zhongyuan, Key Laboratory of Precision Nutrition and Food Quality, Department of Nutrition and Health, China Agricultural University, Beijing 100193, China; 2 College of Food Science and Nutritional Engineering, China Agricultural University, Beijing 100083, China; 3 College of Engineering, China Agricultural University, Beijing 100083, China

**Keywords:** smart nucleic acid hydrogels, biosensors, molecular recognition, stimuli responsiveness, multidimensional application

## Abstract

Smart nucleic acid hydrogels (SNAHs), endowed with stimulus responsiveness, function as programmable molecular switches that can perceive diverse external stimuli and undergo rapid, reversible, and highly specific conformational or performance changes. These dynamic properties have enabled the rational design of biosensors with bionic behaviors, facilitating cascaded “recognition–decision–execution” processes that support advanced biological analysis. Consequently, SNAHs are recognized as a core breakthrough for the next generation of intelligent biosensing units. However, a systematic mapping between SNAH design strategies, specific stimuli, and application fields remains lacking. This review mainly analyzes advances in SNAH-based biosensors over the past five years, proposing flexible and feasible design strategies and key trends in customization. Firstly, we systematically summarize molecular recognition modules involved in the construction of SNAHs, including aptamers, DNAzymes, antibodies, and specific binding peptides. Subsequently, we elaborate on the responses of these modules to external stimuli, so as to further facilitate the signal transduction of signals derived from physical, chemical, and biological sources involving temperature, light, magnetic fields, pH, nucleic acids, proteins, other biomolecules, and pathogens. Additionally, the review outlines the research progress of SNAHs in environmental monitoring, food safety, and medical diagnostics. Finally, we provide an integrated perspective on future opportunities and challenges, highlighting the innovative framework for designing SNAH-based biosensors and offering a practical roadmap for next-generation intelligent sensing applications.

## 1. Introduction

Smart biomaterials capable of precise molecular recognition and stimuli-responsive regulation are revolutionizing the frontiers of biosensing and diagnostics [1]. Although conventional analytical techniques such as enzyme-linked immunosorbent assay (ELISA), polymerase chain reaction (PCR), and high-performance liquid chromatography (HPLC) are well-established, they predominantly depend on complex, non-portable instrumentation and centralized laboratory settings, limiting their applicability for rapid, on-site detection [2,3,4]. Addressing this gap, smart nucleic acid hydrogels (SNAHs) emerge as a distinctive class of materials, characterized by their unique ability to integrate the smart regulatory properties of nucleic acids with the structural versatility and biocompatibility of hydrogel matrices [5]. Unlike conventional hydrogel-based biosensors, which often function as a passive matrix by physically incorporating functional components, SNAHs transcend this role. Crucially, their programmability, rooted in complementary base pairing, allows SNAHs to serve a dual function: serving not only as highly specific recognition elements but also as dynamic three-dimensional network scaffolds with certain mechanical strength [6,7]. This synergy endows SNAHs with precise recognition capability, remarkable structural tunability, reversible sol–gel transition behavior, and efficient signal amplification capabilities [8]. This built-in multifunctionality eliminates the need for bulky peripherals and makes SNAHs uniquely attractive [9,10]. Herein, SNAHs are ideal platforms for developing the next generation of smart biosensors.

The functional core of SNAH-based biosensors is built upon two pillars: specific molecular recognition and efficient signal transduction. To this end, diverse recognition modules, such as aptamers [11], DNAzymes [12], antibodies, peptides [13], and molecular imprinted polymers (MIPs) [14] have been incorporated into SNAHs, thus enabling the specific capture of a broad range of targets from small molecules to pathogens. Moreover, SNAHs are characterized by their versatile responsive mechanisms. They can be engineered to respond to a wide array of external stimuli, including physical (e.g., temperature, light), chemical (e.g., pH, ions), and biological triggers (e.g., nucleic acids, proteins) [15,16]. Importantly, such responsiveness is customizable and controllable, enabling SNAHs to operate as logic-gate systems or multiplexed sensors capable of processing several inputs simultaneously. This responsiveness directly translates molecular binding events into macroscopic, detectable signals, forming the cornerstone of their sensing applications.

Biosensors are increasingly considered for portable on-site detection and point-of-care diagnostics [17]. When the recognition element is combined with responsive mechanisms, biosensors can be effectively applied for analytical purposes. Specifically, in environmental science, SNAHs have been used to detect heavy metals, organic pollutants, and microbial contaminants, offering portable and low-cost alternatives to sophisticated analytical instruments [18]. In the food sector, they enable the sensitive detection of pesticide residues, toxins, and allergens, directly addressing consumer concerns about food quality and safety [19]. In the medical field, SNAH-based systems show promise for the early diagnosis of infectious diseases, detection of cancer biomarkers, and metabolic disorders [20]. Additionally, they hold great potential for coupling therapeutic agent release with diagnostic functions, thereby advancing the emerging concept of theranostics.

**Figure 1:**
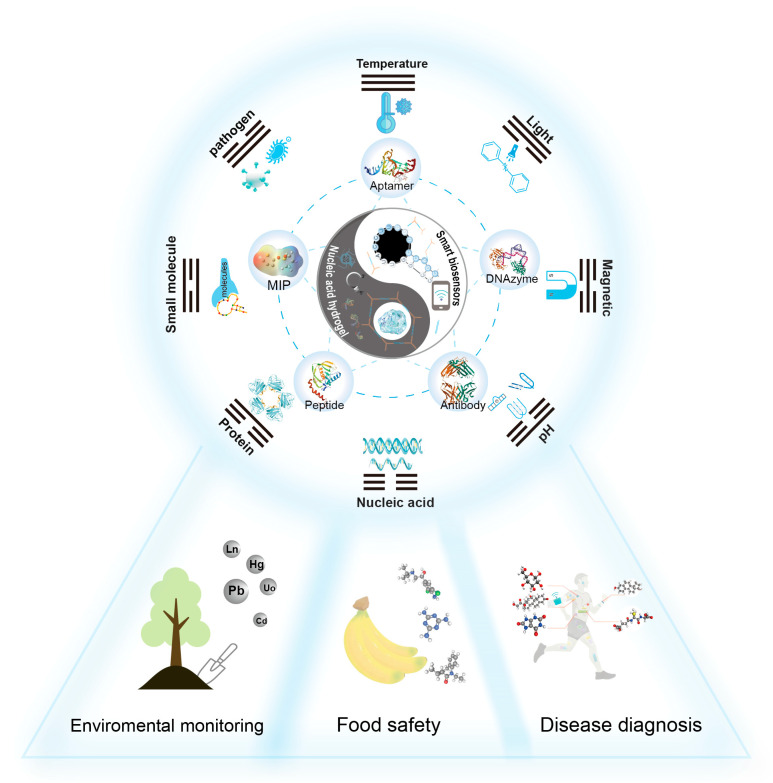
Conceptual framework of nucleic acid hydrogel-based smart biosensors. Molecular recognition elements (aptamer, DNAzyme, peptide, antibody, MIP) interface with stimuli-responsive hydrogel matrices to transduce biochemical signals into detectable outputs, enabling applications in environmental monitoring, food safety, and disease diagnosis.

This review primarily focuses on advancements in biosensor technology, with a particular emphasis on biosensors based on SNAHs (shown in Figure 1). It begins by introducing the integration of recognition elements into SNAHs, followed by a comprehensive classification of stimulus-responsive factors based on eight distinct mechanisms. The review also extensively discusses the applications of SNAHs in key fields, including environmental monitoring, food safety, and disease diagnosis and detection. Additionally, it provides profound insights into future directions for optimizing SNAH-based biosensors to facilitate their practical, real-world use. This work aims to serve as a valuable reference for the design and application of hydrogel-based biosensors.

## 2. Molecular Recognition Elements in SNAHs

Molecular recognition elements demonstrate exceptional selectivity and high affinity toward biological analytes; however, their intrinsic stability limitations significantly impede their practical implementation. To address this challenge, SNAHs, endowed with superior biocompatibility, programmability, and functional integration capacity, provide a robust solution to mitigate the stability issues of molecular recognition elements. This section will elaborate on the key recognition elements, encompassing their fundamental working principles and representative design strategies for their integration into hydrogel networks. These essential components collectively lay a solid foundation for the subsequent signal-responsive functionalities in SNAHs.

### 2.1. Aptamer

Aptamers are short single-stranded oligonucleotides isolated from artificially synthesized random nucleotide libraries via the Systematic Evolution of Ligands by Exponential Enrichment (SELEX) technique [21]. They bind diverse targets with high specificity and affinity, a property facilitated by their facile in vitro synthesis, modification, and sequence design. These attributes enable not only diverse target recognition but also highly sensitive detection, with binding constants ranging from micromolar to picomolar concentrations [22]. Based on the Watson–Crick base-pairing principle, the programmability of aptamers permits their rational integration into SNAHs, primarily through two canonical strategies that underpin the stimulus-responsive behavior of such hydrogels.

**Figure 2:**
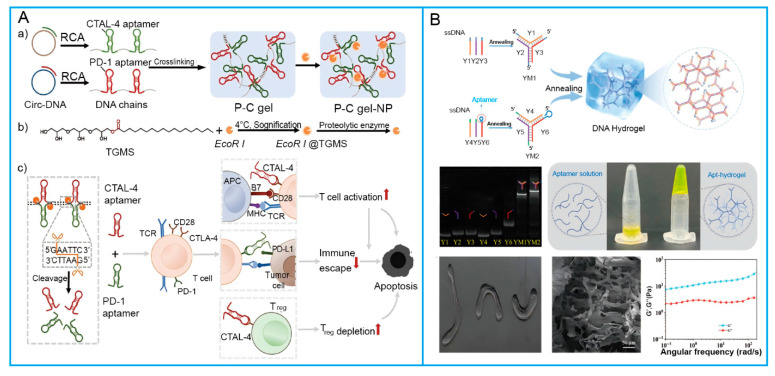
Design of aptamer as recognition element in SNAHs. (**A**). Illustration of preparation process of DNA hydrogel containing poly-aptamers [31]. (a) Synthesis of P-C gel-NP, where CTLA-4 (green) and PD-1 (red) aptamers are conjugated to DNA chains. (b) Fabrication of EcoR I@TGMS nanoparticles. (c) Mechanism of T-cell activation via checkpoint blockade: released aptamers disrupt the interactions between T cells and APCs/tumor cells. (**B**). Schematic illustration of the formation of Apt-hydrogel with two Y-shaped DNA scaffolds [27]. CTLA-4: cytotoxic T lymphocyte antigen 4; PD-1: cell death protein 1; TGMS: Triglycerol monostearate; T cell: T lymphocytes; APC: antigen-presenting cell.

For one, aptamers can act as monovalent crosslinkers [23]. Although this strategy is conceptually elegant, purely DNA-based hydrogels formed via aptamer-complementary strand self-assembly often require large quantities of DNA and may exhibit mechanical instability during cargo release—two factors that limit their practical applicability. Consequently, this approach is less commonly employed in hydrogels. For another, aptamers can function as polyvalent functional nodes, serving as repeating units within the structural scaffold of the hydrogel [24]. Hybrid SNAHs, in turn, can be constructed using such polyvalent functional nodes—with methods including rolling circle amplification (RCA) and branched DNA self-assembly (Figure 2A). RCA is particularly effective in this regard, enabling the enzymatic synthesis of ultralong single-stranded DNA (ssDNA) containing tandem-arrayed aptamer motifs [3]. For example, Yao et al. [25] developed an RCA-based DNA hydrogel integrated with cell-targeting aptamer sequences. This assembly was mediated by the synergistic effects of chain entanglement, multi-primed chain amplification, and hybridization with aptamer-functionalized moieties. Branched crosslinking is another widely used strategy for the self-assembly of DNA hydrogels, typically employing X-shaped, Y-shaped, or T-shaped DNA scaffolds as building blocks [26]. In this approach, specific sticky-end hybridization between complementary DNA backbones and crosslinkers drives the formation of a three-dimensional (3D) hydrogel network. Incorporating aptamer sequences into the sticky ends not only mediates physical crosslinking but also ensures that the aptamers’ target-binding domains are presented in sterically accessible conformations. For example, Chang et al. [27] designed two Y-type DNA building blocks, replacing the “sticky-end” segment in one branch with an aptamer sequence targeting the B subunit of Shiga toxin II (Figure 2B). Likewise, replacing a non-functional palindromic fragment in Y-shaped DNA with the sgc8 aptamer yields a targeted functional DNA hydrogel [28]. Evidently, nucleic acid hydrogels serve as a protective matrix that enhances aptamers’ resistance against nuclease degradation, thereby providing a promising platform for targeted therapy. Beyond DNA aptamers, RNA aptamers have also been exploited for self-assembly into RNA hydrogels, which mitigate the intrinsic chemical instability of RNA and address the limitations of existing RNA-related technologies to a certain extent [29]. For instance, Huang et al. demonstrated that two functional RNA aptamer motifs, M1 and M2, can specifically co-assemble into stable RNA hydrogels. In this system, M1 contains an all-A loop sequence, which is a key motif driving assembly [30].

### 2.2. DNAzymes

DNAzymes are functional nucleic acids with catalytic activity, characterized by both reaction and substrate specificity as well as remarkable selectivity for metal ions. They typically catalyze the hydrolysis of phosphodiester bonds at specific sites within nucleic acid substrates, thereby mediating precise cleavage of nucleic acid strands. Generally, the enzyme chain can quickly cleave the specific site of the substrate chain with the assistance of metal ions or amino acids acting as co-catalysts, while the cleavage efficiency is affected by the DNAzyme concentration and the number of sequences binding on the substrate surface [32]. Therefore, DNAzymes integrated into DNA hydrogels endow biomaterials with catalytic abilities and molecular recognition functions [33,34]. Among them, the G-quadruplex/hemin complex stands out as an artificial DNAzyme with horseradish peroxidase (HRP)-mimicking activity.

**Figure 3:**
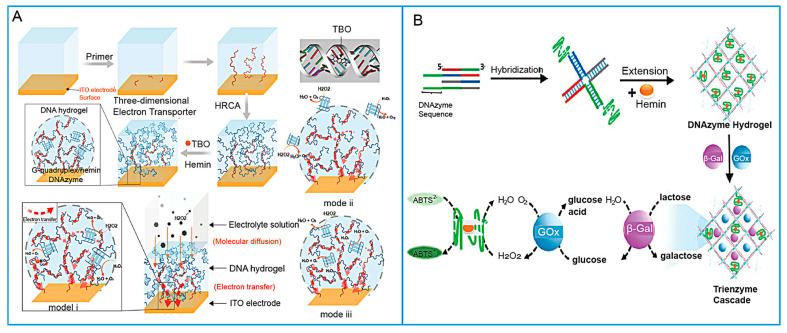
Design of DNAzyme as recognition element in SNAHs. (**A**). DNA hydrogel-based 3D electron transporter fabricated on ITO via HRCA, including three models: i (node-incorporated DNAzyme and TBO-dsDNA docking), ii (DNAzyme in electrolyte), and iii (DNAzyme encapsulated in hydrogel) [36]. (**B**). Self-assembled DNA hydrogel based on enzymatically polymerized DNA for protein encapsulation and implementation of enzyme/DNAzyme hybrid cascade reaction [41]. 3D: three-dimensional; ITO: Indium-tin oxide; HRCA: hyperbranched rolling circle amplification; TBO: toluidine blue O.

The incorporation of DNAzymes into hydrogel is primarily achieved through covalent conjugation or physical encapsulation [35]. This three-dimensional network not only stabilizes the DNAzyme but also provides a favorable microenvironment for its catalytic function and electron transfer. A prevalent strategy leverages in situ synthesis and self-assembly. For instance, Mao et al. [36] predesigned G-rich sequences into a hyperbranched rolling circle amplification (HRCA) template, enabling the spontaneous formation of G-quadruplex structures within the hydrogel scaffold during synthesis (shown in Figure 3A). Upon binding hemin, these structures became functional DNAzymes, enabling a one-pot construction of catalytic hydrogels capable of electron transfer or of generating a colorimetric signal in the ABTS-H_2_O_2_ system [37]. Another common approach involves designing the DNAzyme itself as a stimulus-responsive crosslinker. Jiang et al. [38] engineered a Pb^2+^-binding DNAzyme strand together with a substrate strand containing ribonucleotides. The substrate-mediated base pairing was integrated into the hydrogel as both a responsive unit and a crosslinker. Similarly, DNA hydrogels constructed from three-way junction scaffolds have been functionalized with Ca^2+^-dependent DNAzymes [39] or Zn^2+^-dependent DNAzymes through substrate sequence incorporation [40]. Beyond single-enzyme designs, the intrinsic catalytic properties of DNAzymes have also been harnessed for building multienzyme hydrogel systems. For example, Xiang et al. [41] extended X-shaped DNA motifs using terminal deoxynucleotidyl transferase to incorporate peroxidase-mimicking DNAzymes, thereby developing a self-assembled DNA hydrogel endowed with catalytic functionality (shown in Figure 3B).

Aptamers and DNAzymes exhibit unparalleled programmability and tunability, a core advantage that provides crucial support for constructing precisely engineered hydrogel systems with responsive and self-reporting capabilities. However, these nucleic acid-based molecular recognition elements have two inherent limitations: susceptibility to nuclease degradation and reliance on specific ionic conditions for functionality, which severely restrict their long-term stability and performance in complex biological microenvironments (e.g., body fluids, extracellular matrix).

### 2.3. Antibodies

Antibodies, owing to their high specificity and functional diversity, have been widely applied in bioanalysis and separation technologies [42]. With the continuous advances in antibody production and modification technologies, their integration into nucleic acid hydrogels has shown great promise. As carriers, nucleic acid hydrogels can effectively protect antibodies from protease-mediated degradation and prolong their in vivo activity [43,44]. Simultaneously, conjugating antibodies with nucleic acids to construct multifunctional and highly stable DNA–protein chimeras represents a highly promising strategy [45].

**Figure 4:**
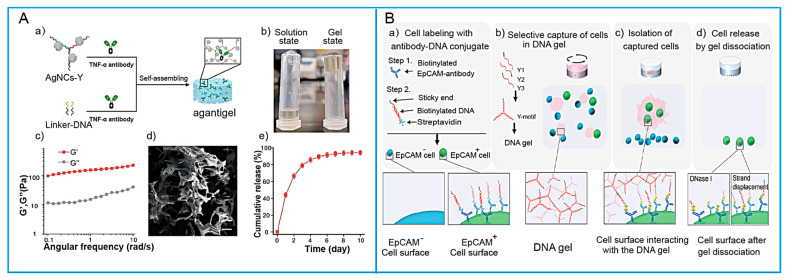
Design of antibodies as recognition element in SNAHs. (**A**). Antibacterial and DNA-based hydrogels in situ block TNF-α to promote diabetic alveolar bone rebuilding [50]. (a) Schematic diagram illustrating the assembly of Agantigel. (b) Photograph of agantigel in solution state (left) and gel state (right). (c) Rheological analysis of Agantigel. (d) SEM image of Agantigel. Scale bar: 100 µm. (e) Release kinetics of TNF-α antibody from Agantigel in vitro. (**B**). Use of DNA gel for capturing circulating tumor cells, with anti-EpCAM antibody as the recognition element (targeting EpCAM-expressing tumor cells, not EpCAM^−^ blood cells) [51]. (a) Labeling EpCAM^+^ cells with anti-EpCAM antibody and sticky-end DNA; (b) engulfing cells into DNA gel; (c) isolating via washing; (d) releasing via DNase I or specific DNA strand-mediated gel dissociation. TNF-α: tumor necrosis factor-alpha; SEM: scanning electron microscopy; EpCAM: epithelial cell adhesion molecule.

The coupling of antibodies and nucleic acids is principally classified into two major categories: covalent and non-covalent construction [46]. Covalent conjugation is typically facilitated by chemical crosslinkers, such as glutaraldehyd and 1-Ethyl-3-(3-dimethylaminopropyl)carbodiimide/N-Hydroxysuccinimide (EDC/NHS) systems. An alternative strategy involves terminal modifications coupled with specific bio-orthogonal reactions, a prominent example being the widely utilized thiol–maleimide reaction. In contrast, non-covalent conjugation predominantly relies on engineered or natural intermolecular affinities. Among these, the biotin–streptavidin system is particularly notable for achieving a high degree of specificity and binding affinity. In this approach, either the antibody or the nucleic acid can be efficiently biotinylated for subsequent binding to a streptavidin-conjugated counterpart. Other non-covalent methods may leverage direct natural interactions between proteins and nucleic acids [47,48]. In general, non-covalent approaches are more straightforward to implement compared with covalent methods. For instance, Zhang et al. [49] designed antibody–DNA chimeras for hydrogel functionalization, in which streptavidin was used to bridge biotinylated antibodies and biotinylated single-stranded nucleic acids. These chimeric systems enable dynamic regulation via DNA-mediated reversible hybridization while retaining specific recognition of target cells. Furthermore, Peng et al. [50] integrated silver nanoclusters (AgNCs) and antibody-based immunotherapy into DNA hydrogels. DNA sequences stabilized on the surface of AgNCs could hybridize with complementary strands, while the other terminus of the DNA sequences was conjugated to tumor necrosis factor-α (TNF-α) antibodies via common biomolecular coupling strategies (shown in Figure 4A). This process led to the formation of a quaternary “AgNCs–Y–L monomer–TNF-α antibody” complex, thereby enabling the controlled release of antibodies from the hydrogel matrix.

As classical biological recognition elements, antibodies exhibit exceptional target affinity and strong biological relevance; however, their practical application remains constrained by several inherent limitations. Specifically, their relatively large molecular size leads to increased mass transfer resistance within hydrogel networks, while the high production cost impedes large-scale and cost-effective implementation. Moreover, their structural fragility under harsh conditions, such as extreme pH, elevated temperature, or exposure to organic solvents, further restricts their stable integration into hydrogel matrices.

### 2.4. Peptides

Peptides, owing to their remarkable molecular recognition ability, have emerged as versatile templates for constructing specific probes to detect diverse biomarkers [52]. A representative example is antimicrobial peptides (AMPs), which function as novel recognition agents and enable the targeted identification of pathogenic bacteria through specific electrostatic interactions with lipopolysaccharides (LPSs) and other negatively charged molecules on bacterial membranes, with their amino acid sequences determining recognition specificity [53,54]. In addition, peptides, due to their small molecular size and high environmental stability, can exhibit exclusive binding affinity to specific materials via conformational adaptability and intermolecular forces. Typical examples include the polystyrene-binding peptide and polypropylene-binding peptide, which facilitate the detection of microplastics [55].

**Figure 5:**
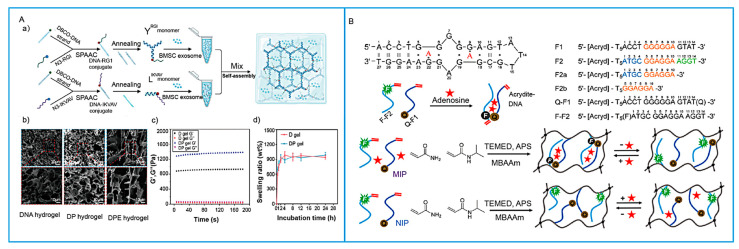
Design of peptide or MIP as recognition element in SNAHs. (**A**). Fabrication and characterization of the programmable DNA–peptide conjugated, exosome-loaded hydrogel [59]. (a) Schematic illustration of the synthesis of DPE hydrogel; (b) SEM images of pure DNA hydrogel, DP hydrogel, and DPE hydrogel; (c) rheological testing of DNA hydrogels (D gel) and DP hydrogels; (d) the swelling character of DNA hydrogels and DP hydrogels in PBS at 37 °C. (**B**). The secondary structure of the adenosine aptamer, sequences of its fragments, the adenosine-binding and fluorescence-quenching mechanism of two fragments, and the preparation of adenosine–MIP and nonimprinted polymers with these aptamer fragments. TEMED and APS were used for initiation, and MBAAm was used as the crosslinker [71]. MIP: molecularly imprinted polymers; APS: ammonium persulfate; TEMED: N,N,N′,N′-tetramethylethylenediamine; MBAAm: methylene bis(acrylamide).

In SNAH-based sensing systems, the recognition capability and amphiphilic nature of peptides synergistically function to reduce nonspecific adsorption by optimizing the distribution of recognition sites [56]. He et al. [57] designed a zwitterionic peptide, which was covalently coupled to the carboxyl groups on a Y-shaped DNA scaffold via an NHS/EDC activation system, and subsequently integrated into the DNA hydrogel network. Similarly, Li et al. [58] constructed DNA–peptide hybrid hydrogels by crosslinking with X-shaped DNA linkers via precise molecular recognition through a Cu^+^-catalyzed click chemistry reaction. Notably, click chemistry exhibits mild reaction conditions, no byproducts, and nanometer-scale modification precision. Wei et al. [59] further employed strain-promoted azide–alkyne cycloaddition to couple chemistry-modified branch-shaped DNA with azide-functionalized peptides at 4 °C, yielding peptide-functionalized DNA monomers (Figure 5A). Huang et al. [60] introduced both M6P molecules and VEGFR-targeting peptides into the Y-motif DNA scaffold via click chemistry, establishing a “dual-recognition site” system to address the limitation of insufficient binding strength from single-site recognition. To overcome the intrinsic challenge of poor binding efficiency, Yin et al. [61] synthesized a peptide–tFNA structure by conjugating peptides onto tetrahedral framework nucleic acids (tFNAs), where the 3D rigid geometry of tFNAs fixed peptides at specific spatial positions, thereby enhancing stability. Meanwhile, Obuobi et al. [62] proposed a distinct strategy by exploiting the electrostatic adsorption between AMPs and Y-shaped DNA monomers to construct peptide-loaded DNA hydrogels.

Peptides offer an attractive compromise between synthetic versatility and biological recognition, combining ease of chemical modification, favorable biocompatibility, and cost-effectiveness. These attributes position peptides as valuable complementary recognition units within SNAH-based biosensing frameworks. However, despite their structural adaptability, peptide-based systems often exhibit inherently lower selectivity toward small-molecule targets and weaker binding affinities—typically within the micromolar (μM) range—compared with the nanomolar (nM) or even picomolar (pM) affinities of nucleic acid- or antibody-based receptors. This limitation becomes particularly pronounced in scenarios demanding ultra-high specificity or quantitative discrimination among structurally similar analytes.

### 2.5. MIPs

MIPs, as a class of synthetic receptors with “molecular memory” characteristics, can form specific recognition sites through template-directed polymerization [63,64]. These recognition sites are complementary to the spatial conformation and chemical properties of target molecules. Owing to their high stability, low cost, and customizability, MIPs have emerged as important recognition units in nucleic acid hydrogels for biosensing [65].

The recognition mechanism of MIPs is selective binding with target molecules through non-covalent interactions or reversible covalent interactions [66]. Their recognition performance can be precisely tuned by rational selection of functional monomers, adjustment of crosslinking density, and optimization of imprinting strategies [67]. In SNAH systems, MIPs are generally integrated in two modes: for one, dispersing MIPs as functional fillers within the hydrogel network, in which their porous structures and specific cavities provide additional recognition sites [68]; for another, incorporating nucleic acid molecules (such as aptamers or DNA fragments) as imprinting templates or functional monomers to construct “nucleic acid–MIP” hybrid systems that achieve synergistically enhanced recognition performance [69]. In recent years, researchers have attempted to further improve the specificity and selectivity of MIPs by introducing functional units such as aptamers or boronic acid groups. Li et al. [70] employed an acrylamide-modified full-length adenosine aptamer as a macromonomer to copolymerize with 3-acrylamidophenylboronic acid, thereby combining the aptamer’s high specificity for the adenosine base with the covalent interaction between boronic acid and the cis-diol of ribose. This strategy led to the construction of a dual-recognition aptamer–MIP hydrogel. This hydrogel effectively overcame the inability of conventional adenosine aptamers to distinguish adenosine from structurally similar nucleosides (e.g., deoxyadenosine, cytidine). Moreover, the dual-recognition aptamer–MIP hydrogel maintained selective recognition in 5% fetal bovine serum, demonstrating superior performance compared to single aptamer systems. Similarly, Zhang et al. [71] used an acrylamide-modified adenosine aptamer fragment as a macromonomer to copolymerize with acrylamide and N-isopropylacrylamide (shown in Figure 5B). Through molecular imprinting, the spatial conformation of the fragment was fixed and its binding ability restored. This approach significantly enhanced the binding affinity of MIPs.

As fully synthetic molecular recognition elements, MIPs have attracted increasing interest for their remarkable mechanical stability, chemical resilience under harsh conditions, and scalability in production. These features make MIPs particularly appealing for constructing durable and cost-effective biosensing interfaces. Nonetheless, intrinsic limitations remain unresolved: incomplete template removal and the non-uniform spatial distribution of binding sites often compromise recognition fidelity, leading to slower binding kinetics and reduced specificity compared with biomacromolecular receptors. These shortcomings pose persistent challenges for the development of MIP-based sensors capable of rapid, high-fidelity detection.

From a performance optimization standpoint, hybridization strategies that combine distinct classes of molecular recognition elements are increasingly recognized as an effective means to transcend the intrinsic limitations of individual components (Table 1). Representative examples include aptamer–MIP composites and peptide–DNA hybrid hydrogels. These hybrid constructs not only reinforce signal reliability, reusability, and operational stability but also provide a conceptual framework to reconcile the longstanding trade-off between sensitivity and robustness. Such integrative designs pave the way for the next generation of biosensing platforms capable of reliable performance in complex clinical and environmental matrices.

## 3. Stimulus Response Mechanisms and Signal Transduction in SNAHs

Bridging molecular recognition and signal output, programmable structural changes are the core of SNAHs. These changes are engineered to respond to specific stimuli via two main paradigms. For one, physicochemical stimuli (e.g., pH, temperature) can directly induce structural transitions by exploiting the innate properties of nucleic acids (e.g., i-motif folding). For another, and more prominently, the binding or catalytic events involving recognition modules (aptamers, DNAzymes, antibodies, etc.) trigger pre-programmed structural reconfigurations, such as strand displacement or cleavage, leading to a macroscopic gel–sol transition. This section will detail how these mechanisms effectively convert a wide range of stimuli into measurable signals.

### 3.1. Temperature-Responsive SNAHs

Temperature-sensitive hydrogels are a representative category of smart responsive materials, whose physical properties can undergo reversible transitions upon exposure to external temperature changes [72]. Critically, temperature serves as a powerful switch to control molecular recognition within these hydrogels. By precisely manipulating the hybridization kinetics of nucleic acids and the phase behavior of functional polymers, temperature can act as a powerful switch to control the accessibility, affinity, and activity of embedded molecular recognition elements, thereby programming the sensing behavior of the hydrogel.

Since the stability of DNA duplexes and the folding of aptamers are highly temperature-dependent, the binding events can be designed to occur only within a specific temperature window. An elevated temperature can denature the duplexes that “lock” an aptamer in an inactive state, thereby activating its binding capability [73]. Beyond nucleic acids themselves, thermosensitive polymers like poly(N-isopropylacrylamide) (pNIPAM) can amplify molecular recognition through macroscopic volume transitions. pNIPAM swells below its lower critical solution temperature (LCST) and shrinks above it due to hydration changes. This phase transition can mechanically transduce a molecular binding event into a pronounced physicochemical output [74]. For example, Chen et al. [75] designed pNIPAM-DNA hydrogels functionalized with aptamers specific to amyloid-β oligomers that can capture target molecules. In this system, the aptamer-mediated capture of the target is not the final signal but the trigger that initiates a downstream enzymatic reaction; the temperature-responsive hydrogel matrix then acts as a confined reactor, where the LCST behavior can potentially enhance the local concentration of reactants and the efficiency of the signal amplification process. Beyond intrinsic thermal response, the integration of photothermal nanomaterials allows for the remote and spatially precise control of molecular recognition. Moreover, the integration of gold nanoparticles (AuNPs) and gold nanorods (AuNRs) endows hydrogels with photothermal responsiveness through the localized surface plasmon resonance effect [76]. Upon laser irradiation, the localized heat generated by AuNPs/AuNRs induces the dissociation of DNA duplexes, thereby regulating hydrogel stiffness [77]. Wang et al. [78] demonstrated that the laser-induced heating of AuNPs could dissociate DNA crosslinkers, effectively using temperature as a tool to reconfigure the hydrogel network and thus control the diffusion and access to molecular recognition elements. The finding by Wang et al. [79] on the “breathing” dynamics of surface-grafted DNA strands further suggests that local thermal fluctuations can fine-tune the presentation and binding affinity of ligands, offering a nanoscale mechanism to modulate recognition kinetics.

In summary, temperature-responsive SNAHs exploit thermal energy to gate, activate, and enhance molecular recognition events, making these hydrogels powerful platforms for applications requiring precise temporal and spatial control.

### 3.2. Light-Responsive SNAHs

**Figure 6:**
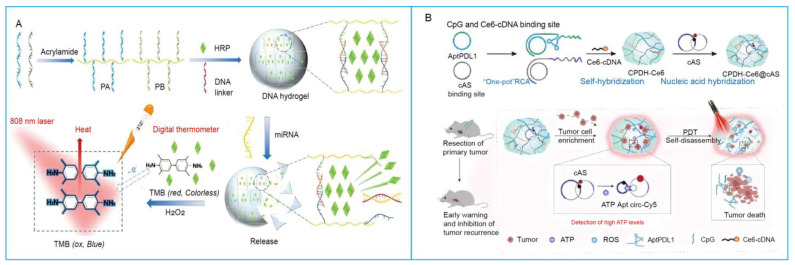
Light-responsive SNAHs. (**A**). Schematic illustration of the synthesis of stimuli-responsive DNA hydrogels and photothermal sensing detection of miRNAs based on HRP-mediated TMB-H_2_O_2_ colorimetric system [90]. (**B**). Cyclic DNA templates containing complementary sequences of PD-L1 aptamer and CpG, along with photosensitizer Ce6-cDNA, were used for the synthesis of photoresponsive immunomodulatory hydrogels via a one-pot RCA reaction [88]. TMB: 3,3′,5,5′-tetramethylbenzidine; RCA: rolling circle amplification.

Light offers superior spatiotemporal precision as a non-invasive stimulus for controlling SNAHs [80], and it is usually reversible. The molecular recognition in these systems is uniquely gated by light, primarily through the integration of photoresponsive units that act as molecular switches or triggers (Figure 6A). Typical photoisomerizable molecules like azobenzene and arylazopyrazole [81] are capable of interacting with DNA, thereby enabling them to finely regulate the stability of double-stranded nucleic acids.

In common design strategies, azobenzene undergoes conformational transitions under irradiation with different wavelengths, thereby inducing the rupture and reformation of crosslinking points and driving reversible transitions between the hydrogel and solution states [82]. For example, Peng et al. [83] employed trans-azobenzene units to stabilize the double-stranded structure, whereas cis-azobenzene destabilized it, thus enabling light-induced reversible transitions. In general, most light-responsive DNA hydrogels are introduced by polymer matrices and synergistically stabilized by permanent crosslinking units, such as boronate ester/glucosamine and trans/cis photoisomerizable nucleic acid bridges [84]. Liu et al. [85] integrated both glucosamine-boronate ester bonds and trans-azobenzene-stabilized DNA duplex bridges as cooperative crosslinkers. Upon irradiation at 365 nm, azobenzene isomerizes from the trans to the cis form, resulting in hydrogel softening; exposure to light above 420 nm reverses the isomerization, restoring the hydrogel’s stiffness. In addition to these direct structural reconfigurations, light can also be harnessed to trigger more complex functional responses within SNAHs. For instance, nanomaterials like quantum dots (QDs) can be integrated to serve dual purposes. Upon light irradiation, QDs can act as efficient nanoscale heaters due to their excellent photothermal conversion efficiency, inducing localized heating that leads to volumetric changes in the hydrogel, such as swelling or collapse of the network [86]. Simultaneously, their strong photostability and tunable emission allow them to function as robust optical transducers, providing a real-time fluorescence readout of these physicochemical changes [87]. This dual functionality enables a self-reporting system where the actuation (physical response) and the sensing (optical signal) are intrinsically coupled. Beyond conformational switching, light can trigger a cascading amplification system (initiated by molecular recognition) by generating reactive oxygen species (ROS), thereby enabling the integration of light into the system. Wang et al. [88] designed a DNA hydrogel where the primary molecular recognition of adenosine triphosphate (ATP) not only generated a fluorescence signal but also primed the system for a secondary, light-driven response (Figure 6B). Specifically, the ATP-binding event localized the photosensitizer Ce6. Subsequent localized irradiation then served as an orthogonal stimulus that activated Ce6, generating ROS. This cascade transduced and amplified the initial molecular recognition event into massive backbone cleavage, network disruption, and controlled drug release for synergistic therapy. Furthermore, research on light-responsive DNA hydrogels has been extended to near-infrared (NIR) light-triggered photothermal effects. Liu et al. [89] reported an injectable NIR-responsive hydrogel based on DNA-AuNP hybrid structures, where gold nanoparticles efficiently absorbed light energy via surface plasmon resonance (SPR) and converted it into thermal energy, thereby achieving controlled photothermal regulation of the hydrogel.

### 3.3. Magnetic-Responsive SNAHs

The responsive performance of magnetic hydrogels is highly dependent on the design and parameters of the applied magnetic field [91]. Previous studies have demonstrated two primary strategies for constructing magneto-responsive DNA hydrogels [92]. For one, embed magnetic nanoparticles (MNPs) directly into the DNA hydrogel network. For another, integrate MNPs with DNA strands or their complementary sequences via chemical conjugation or DNA hybridization [93,94]. The incorporation of MNPs not only imparts remote manipulability under external magnetic fields but also significantly enhances the structural stability of the hydrogel. In practice, Ma et al. [95] designed a magneto-responsive DNA hydrogel in which DNA-modified MNPs were hybridized with Y-shaped DNA to achieve a hybridization-driven embedding. Upon application of an external magnetic field, the hydrogel exhibited multidirectional deformation and even remote locomotion along the substrate surface, offering novel strategies for cell culture and biomedical applications. Similarly, Tang et al. [96] employed RCA to generate ultralong ssDNA with capture sequences, which were subsequently hybridized with complementary strand-modified MNPs to fabricate a magnetic DNA hydrogel-based soft robot. This system could be magnetically guided to specific sites for controlled payload release, highlighting its potential as an intelligent tool for live-cell transport. Furthermore, Yao et al. [97] developed a magnetic hydrogel employing amino-functionalized Fe_3_O_4_ nanoparticles with RCA products via physical crosslinking, enabling magnetically controlled drug delivery and on-demand release, providing a promising strategy for precision medicine.

While traditional magnetic SNAHs primarily utilize magnetic nanoparticles for physical remote control, the integration of molecular recognition elements transforms them into precisely targeted and autonomously responsive systems. The future of magnetic-responsive SNAHs lies in moving beyond such simple physical remote control toward systems where magnetic fields and molecular recognition operate in concert. This synergy will enable sophisticated functions, such as autonomous target-driven drug release, highly sensitive remote biosensing, and real-time reporting of biochemical activity, thereby paving the way for next-generation precision medicine platforms.

### 3.4. pH-/Ion-Responsive SNAHs

The programmable secondary structures of nucleic acids confer an intrinsic sensitivity to the chemical environment, particularly to protons and specific metal ions [98]. This allows pH and ions to act as fundamental inputs for molecular recognition in SNAHs, triggering precise structural reconfigurations and signal transduction. Protons, as the primary regulators of pH, together with other metal ions, participate in the modulation of the acid–base equilibrium in solution, thereby influencing DNA conformations and hydrogel properties [99]. Among the typical motifs, cytosine-rich sequences can fold into i-motif structures under acidic conditions, while guanine-rich sequences fold into G-quadruplexes in the presence of K^+^ [100]. The formation of i-motif structures relies on the partial protonation of cytosine residues (pH = 6.5), which form C:C^+^ base pairs through Hoogsteen interactions and assemble into a stable tetraplex crosslinking unit. Conversely, adenine residues can be protonated in strongly acidic environments, forming A-motif structures [101]. These two types of structures often exhibit synergistic effects in hydrogels, and their acid–base transition processes have been utilized to simulate structural stability under different gastrointestinal environments [102].

**Figure 7:**
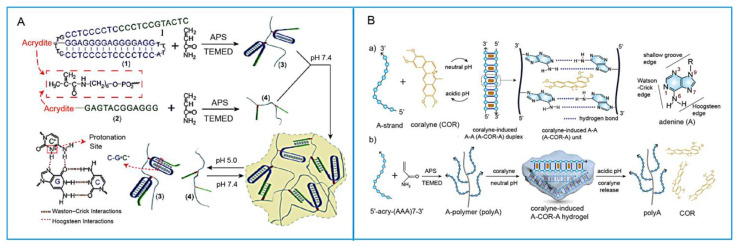
pH-/Ion-Responsive SNAHs. (**A**). Synthesis of the DNA chains capable of undergoing pH-stimulated hydrogel formation and dissociation [104]. (1): Acrydite nucleic acid. (2): Acrydite nucleic acid complementary to (1). (3): Linear copolymers formed by the copolymerization of acrylated nucleic acids (1) and acrylamide. (4): Linear copolymer formed by the copolymerization of acrylated nucleic acid (2) and acrylamide. (**B**). Assembly of oligoside adenine with coralyne to pH-responsive DNA hydrogel [105]. (a) COR induces the assembly of A-strand into antiparallel A-COR-A duplex at neutral pH, with dissociation triggered under acidic pH; (b) Acrydite-modified A-strands copolymerizes with acrylamide to form polyA, which self-assembles into A-COR-A DNA hydrogel at neutral pH. Under acidic conditions, COR release triggers hydrogel-to-liquid transition. COR: coralyne.

DNA triplex structures provide another pathway for pH responsiveness. At pH 5.0, protonated C-G·C triplexes stabilize acidic conformations, while T-A·T triplexes remain stable under neutral conditions but dissociate into T-A duplexes under alkaline conditions (pH 10.0) due to thymine deprotonation [103]. Ren et al. have utilized this property to construct switchable triplex-based DNA hydrogels [104] (Figure 7A). Furthermore, duplex-based systems can also exhibit pH-responsive behavior. For example, adenine strands can assemble with the small-molecule drug coralyne (COR) to form a hydrogel stable at neutral pH. Lee et al. [105] found that upon pH variation, the instability of A-COR-A units leads to hydrogel disassembly accompanied by COR release, thereby enabling controlled drug delivery (Figure 7B).

In addition to proton-mediated mechanisms, metal ions also regulate DNA secondary structures, imparting ion-specific responsiveness to hydrogels. For instance, Pb^2+^ can induce DNAzyme folding into G-quadruplex structures, triggering sol–gel transitions [106]. Hg^2+^ can interact with thymine residues to form T-Hg^2+^-T hairpins [107], and Ag^+^ can coordinate with cytosine bases to form C-Ag^+^-C base pairs, leading to hydrogel contraction [108]. Notably, ZnO nanoparticles have been introduced as pH-responsive nanomaterials, which remain stable under neutral conditions but rapidly dissolve under acidic environments to release Zn^2+^ [109,110]. Yao et al. [111] demonstrated that pH-responsive DNA hydrogels, prepared by crosslinking carboxylated Y-DNA probes with aminated ZnO NPs, disintegrated under acidic conditions due to ZnO dissolution, releasing DNA probes and enabling sensitive miRNA detection through ratiometric fluorescence signals.

### 3.5. Nucleic Acid-Responsive SNAHs

Strand displacement reactions trigger a rapid cascade effect from assembly to disassembly in DNA hydrogels [112]. In this process, target nucleic acids competitively hybridize with DNA linkers in the hydrogel network, forming more stable base pairs and releasing the crosslinking chains, thereby inducing hydrogel degradation [113]. Lin et al. [114] first demonstrated this strategy by constructing DNA–polyacrylamide hydrogels crosslinked with double-stranded DNA, which dissociated upon the introduction of removal strands. Building on this concept, Ho et al. [115] designed adenine-rich oligonucleotides to replace melamine in T-MA-T duplexes, forming stable A-T structures that functioned as reversible “nanobridges” for hydrogel assembly and disassembly. Nucleic acid-triggered strand displacement can also be coupled with specific capture probes to achieve precise detection [116]. For instance, Guo et al. [117] constructed an aptamer-based hydrogel system responsive to the PML/RARα fusion gene. In this design, a PML/RARα-specific capture probe was immobilized on the gold surface of an SPR chip, while aptamers embedded in the hydrogel enriched streptavidin. The presence of the target nucleic acid triggered hydrogel rearrangement, leading to changes in molecular mass and local refractive index, thereby amplifying the detection signal.

RNA can also act as a trigger for hydrogel disassembly. Liu et al. [90] reported a DNA–polyacrylamide hydrogel responsive to microRNA (miRNA), where DNA linkers complementary to the target miRNA mediated gel collapse. The hybridization of miRNA with the linkers disrupted polyacrylamide crosslinks, leading to hydrogel disassembly and the release of encapsulated HRP. The released HRP catalyzed H_2_O_2_ decomposition, producing a photothermal signal under near-infrared irradiation and thereby enabling miRNA detection. Beyond this, strand displacement-activated DNA hydrogels have shown strong potential in miRNA biosensing [118]. For instance, a ratiometric fluorescence method employing Cy5-labeled DNA was applied to detect miRNA-21. Likewise, Chang et al. [119] proposed that miRNA-141 competitively displaces the bridge DNA within the hydrogel microcapsule shell, causing the capsule to disassemble and release quantum dots, which in turn enhances the fluorescence signal.

### 3.6. Protein-Responsive SNAHs

Protein-responsive SNAHs display a smart response to target proteins through specific molecular recognition, thereby inducing structural transformations or functional outputs [120]. Multiple strategies have been proposed surrounding aptamers and functional nucleases (e.g., Cas enzymes in the CRISPR-Cas system) [121,122]. For instance, Zhang et al. [123] reported a competitive binding regulation strategy, in which complementary DNA (cDNA) hybridizes with aptamers immobilized within the hydrogel, occupying their protein-binding sites and thereby inhibiting target protein capture. External stimuli (e.g., light or specific nucleic acid strands) can then induce cDNA dissociation, usually reversible.

Aptamers, as recognition elements, induce mainly protein responsiveness in SNAHs [124]. Sun et al. [125] constructed a mucin 1 protein (MUC1) detection platform by integrating DNAzyme and its substrate strand into a hydrogel matrix. Upon MUC1 binding, the DNAzyme is activated to cleave the substrate strand, and the resulting fragments induce changes in nanopore current, thereby achieving ultrasensitive detection of MUC1 or circulating tumor cells. Similarly, Ji et al. [126] designed a conformation-switching hydrogel, in which a hairpin aptamer specific for carcinoembryonic antigen was covalently grafted onto a poly(methacrylic acid) backbone via amide bonds. The binding of antigen to the aptamer triggered the opening of the toehold region, altering molecular conformation and inducing macroscopic responses such as hydrogel swelling or degradation.

SNAHs constructed through enzyme-specific recognition enable dynamic control over material properties and versatile signal transduction, leveraging the high specificity and catalytic efficiency of enzymatic reactions [127]. Gao et al. [128] designed a DNA tetrahedral framework hydrogel with edge chains containing Dam methyltransferase recognition sites. Dam-mediated adenine methylation altered DNA intermolecular interactions, modulated crosslinking density, and enabled signal readout through a personal glucose meter, thereby achieving specific detection of Dam methyltransferase. Furthermore, Gayet et al. [129] incorporated the CRISPR-Cas system into responsive hydrogels by using ssDNA bridge strands containing Cas12a-crRNA target sequences to crosslink DNA–polyacrylamide hydrogels.

### 3.7. Small Molecule-Responsive SNAHs

Small-molecule-responsive SNAHs generally exploit the specific recognition ability of aptamers to capture small molecular analytes, such as antibiotics (e.g., kanamycin, tetracycline) and biotoxins (e.g., cocaine, ATP) [130,131,132]. As molecular switches, aptamers trigger allosteric effects upon interacting with target molecules within the confined three-dimensional space of the hydrogel. These conformational changes are further transduced into amplified signals through fluorescence, spectroscopy, or electrochemical readouts, thereby enabling highly sensitive detection [133].

**Figure 8:**
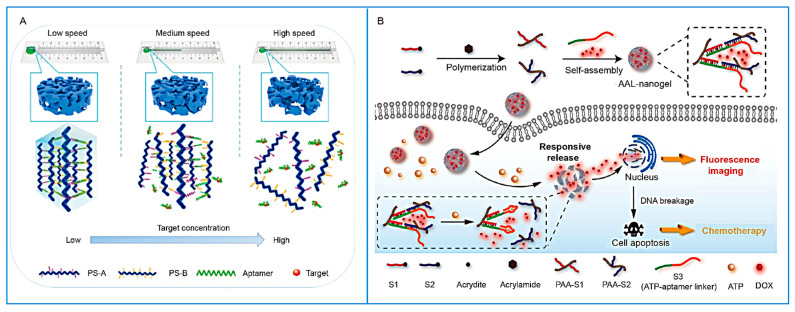
Small molecule-responsive SNAHs. (**A**). Schematic illustration of the working principle of the CSDR-Sensor [137]. The addition of cocaine molecules causes a certain degree of hydrogel degradation, which in turn alters the gel’s permeability in the capillary tube and regulates capillary action by modifying membrane permeability. (**B**). Self-assembled ATP-responsive DNA nanohydrogel for specifically activated fluorescence imaging and chemotherapy in cancer cells [139]. CSDR: capillary self-driven regulator sensor; ATP: adenosine triphosphate.

In the antibiotic detection context, Chen et al. [134] developed a kanamycin aptamer-based sensor employing surface-enhanced Raman scattering (SERS). Upon binding to kanamycin, the aptamer underwent a conformational change that released the primers, thereby initiating RCA and forming a DNA hydrogel network embedded with gold nanoparticles and magnetic beads. The quantification of hydrogel formation enabled the real-time and highly sensitive detection of kanamycin [135]. Moreover, signal amplification strategies based on DNA walkers have also been applied to antibiotic detection. For instance, Sun et al. [136] designed a DNA walker system using streptavidin-coated magnetic beads functionalized with hairpin DNA. In the presence of cephalosporin antibiotics, the walking strand was activated to trigger nuclease cleavage, cutting FAM-labeled hairpin structures and generating fluorescence signals, thereby enabling highly accurate detection. In the toxin detection domain, Li et al. [137] proposed a capillary self-driven regulator sensor capable of converting cocaine-induced microstructural changes in DNA hydrogels into macroscopic flow-rate signals (Figure 8A). Mechanistically, cocaine binding to the aptamer increases the permeability of hydrogel membranes, altering the flow duration of sample solutions within capillaries and thus enabling quantitative detection based on time parameters. In the ATP-responsive system, ATP aptamers specifically bind to ATP molecules, competitively dissociating complementary hybridized strands and thereby regulating the conformation and crosslinking density of the hydrogel [138]. This mechanism enables in situ and hierarchical modulation of the hydrogel’s mechanical properties. Xu et al. [139] designed a nanogel, for which, in the presence of ATP, aptamer binding triggers the disassembly of nanogels and the release of the encapsulated anticancer drug doxorubicin (DOX), enabling the dual functions of chemotherapy and fluorescence imaging in cancer cells (Figure 8B).

### 3.8. Pathogen-Responsive SNAHs

The rapid and specific detection of pathogens such as bacteria and viruses is critical for public health [140]. Pathogen-responsive SNAHs address this need by integrating specific molecular recognition elements for whole pathogens or their unique components, and transducing binding events into macroscopic signals through programmable structural changes in the hydrogel network.

For bacteria-responsive systems, the molecular recognition primarily targets surface antigens or employs charge interactions, leading to therapeutic or diagnostic outputs. Obuobi et al. [62] achieved this through electrostatic interactions between polyanionic DNA nanostructures and cationic AMPs, which could respond to pathogenic *Staphylococcus aureus* infection and exhibited notable antibacterial potential. In the context of detection, nucleic acid aptamers are often employed as crosslinkers within the hydrogel while simultaneously functioning as signal transducers. For instance, Yu et al. [141] modified a Vibrio parahaemolyticus (V.P.) aptamer onto the cap of an Eppendorf tube and, by employing ATP as a signal mediator, designed an intelligent aptamer–hydrogel sensor capable of quantitatively detecting V.P.

Virus-responsive SNAHs predominantly rely on the recognition of unique viral nucleic acid sequences, which are then amplified to induce structural changes in the hydrogel for ultrasensitive detection [142]. Na et al. [143] employed viral nucleic acids as trigger signals and achieved RCA within microfluidic channels, where the amplified DNA hybridized with dumbbell-shaped padlock probes to form DNA hydrogels, ultimately leading to channel blockage and enabling the visual identification of viral presence. Similarly, Kin et al. [144] reported a DNA hydrogel detection platform based on RCA that enabled highly sensitive detection of SARS-CoV-2 within 15 min, with a limit of detection (LOD) of 0.7 aM, comparable to conventional PCR. Furthermore, Jiao et al. [145] use SARS-CoV-2 nucleic acids as triggering signals. An EXPAR–DNAzyme cascade was integrated to cleave L-DNA crosslinkers. This molecular recognition event was effectively converted into macroscopic readouts such as colorimetric and thermal signals [9].

## 4. Application of SNAHs in Biosensors

Given the exceptional molecular recognition capabilities and stimulus-responsive properties of SNAHs described earlier, this chapter will thoroughly explore their extensive applications in biosensors for human health.

### 4.1. Environmental Monitoring

Heavy metal ions and organic pollutants in the environment are resistant to microbial degradation and tend to accumulate persistently in organisms and ecosystems [146]. Through strong interactions with biomacromolecules such as proteins, they induce significant toxicity, thereby posing serious threats to both ecological balance and human health [147]. Consequently, the development of rapid and highly sensitive detection technologies for heavy metal ions in water has long been a central challenge in environmental monitoring. Conventional approaches, such as inductively coupled plasma mass spectrometry and chromatography, offer high sensitivity but suffer from limitations including operational complexity, high instrumentation costs, and strict laboratory requirements, making them unsuitable for rapid on-site screening [148,149]. In recent years, SNAHs-based biosensing systems have emerged as a promising alternative. They harness functional nucleic acids, primarily aptamers or DNAzymes, that undergo conformational changes upon specific binding to target ions, subsequently transducing the interaction into measurable physical signals (e.g., fluorescence, colorimetric, or electrochemical outputs) [150]. Furthermore, embedding recognition nucleic acids into three-dimensional hydrogel networks enables the construction of intelligent responsive platforms that combine molecular recognition with intrinsic signal amplification.

Representative analytes of environmental concern include Pb^2+^, Hg^2+^, UO_2_^2+,^ and Cd^2+^. For instance, Pb^2+^-specific DNAzymes activate catalytic cleavage of substrate strands upon binding, leading to hydrogel network disassembly and release of encapsulated fluorophores, thereby enabling trace-level detection. In Hg^2+^ detection, early studies exploited thymine-rich DNA sequences that form T-Hg^2+^-T coordination structures for selective recognition [151,152]. However, interference from complex sample matrices remains a major obstacle to ultratrace detection. To address this challenge, Pi et al. [153] integrated nucleic acid recognition with the diffusive gradients in thin films (DGT) technique, creating a DNA-DGT sensor. This device integrates a DNA-functionalized hydrogel with high load-carrying capacity into the DGT, enabling the complete process of mercury ion transport from diffusion to binding and subsequent encapsulation within the protective structure. Leveraging specific interactions, the system effectively excludes competing ions, allowing direct quantitative readout via fluorescence without complex sample pretreatment. This facilitates rapid on-site monitoring of Hg^2+^. Nevertheless, the reliance on gel imaging systems for signal readout still limits the portability and device integration. To simplify detection workflows and reduce costs, Chu et al. [154] reported a one-step pure DNA hydrogel sensor. In this design, Pb^2+^ activates DNAzyme cleavage of substrate strands, inducing hydrogel collapse and releasing DNA fragments that can be directly monitored by absorbance. This platform achieved a detection limit of 7.7 nM, with a linear response in the range of 0–500 nM, while eliminating the need for additional labeling, thus offering operational simplicity and cost efficiency. For uranyl ion (UO_2_^2+^) detection, SERS has been employed for signal amplification. He et al. [155] developed a flexible DNAzyme-based SERS hydrogel sensor, in which UO_2_^2+^ induces substrate cleavage and triggers the release of rhodamine B (RhB), producing the first stage of signal amplification. Subsequently, RhB molecules are captured by Ag-NPs@PAN films, resulting in further SERS enhancement as a second amplification stage. This dual-amplification strategy successfully integrates the flexibility of DNA hydrogels with SERS technology, providing a robust platform for the ultrasensitive detection of toxic metals in harsh environments. Given the chemical complexity of environmental pollutants, the development of multiplexed and universal detection platforms is highly desirable. Liu et al. [156] employed a layer-by-layer assembly strategy to fabricate ultrathin functional films enriched with AuNPs. In the presence of Pb^2+^ or UO_2_^2+^, activation of the corresponding DNAzyme triggered substrate cleavage, leading to film degradation and release of AuNPs, which produced visually detectable colorimetric responses. By programming different DNA sequence codes, this platform achieved specific recognition of multiple metal ions, thereby enhancing both detection sensitivity and device stability, as well as storage robustness.

Beyond heavy metals, NAH-based strategies have also been extended to the detection of organic pollutants. Liu et al. [157] designed a dual-mode colorimetric/electrochemical sensor integrated into a microfluidic chip, employing a ferrocene-labeled malathion aptamer (MAF) as both recognition unit and hydrogel crosslinker. Specific binding between malathion and MAF triggered hydrogel disassembly, releasing encapsulated AuNPs to produce colorimetric signals, while simultaneously reducing electrochemical currents for quantitative readout. This system demonstrated portability, rapid detection, and no requirement for sample pretreatment. Moreover, with the rising concern over microplastic pollution, DNA aptamers specific to polyvinyl chloride and polystyrene have recently been identified [158], providing a foundation for developing NAH-based microplastic sensors.

The applications of SNAHs in biosensors for environmental monitoring are shown in Table 2. To further improve detection performance, additional amplification strategies have been incorporated into NAH sensing platforms, including capillary flow regulation [38], CRISPR/Cas systems [159], and metal–organic frameworks (MOFs) [160]. These innovations not only enhance sensitivity and resistance to interference but also facilitate the evolution of NAH sensors from single-analyte detection toward multiplexed, on-site environmental monitoring, highlighting their strong potential for practical applications.

### 4.2. Food Safety

Food safety has been an enduring core topic of public health. The presence of pesticide residues, mycotoxin contamination, pathogenic microbes, and illegal food additives [162] renders strict screening, early warning, and prevention necessary. Conventional detection methods, like liquid chromatography (LC), gas chromatography (GC), and fluorescence detection–mass spectrometry (FLD-MS), offer high accuracy but face two critical limitations. For one, they require tedious and time-consuming sample pretreatment. For another, they rely on large-scale, non-portable laboratory equipment [163,164]. This makes them unable to meet real-world needs for rapid and on-site detection. Against this demand for more efficient technologies, SNAHs emerge as a promising solution [165]. Their core advantages directly address conventional methods’ shortcomings: good bearing capacity, facile functionalization, and diverse signal transduction [166]. Collectively, these traits make SNAHs an ideal platform for rapid safety monitoring.

Mycotoxins are secondary metabolites produced by specific fungi, including aflatoxins (AFs), ochratoxin A (OTA), zearalenone (ZEN), fumonisin B1 (FB1), T-2 toxin, etc. [167]. Even at trace levels, these toxins exert pronounced toxicity, causing severe damage to the liver, kidneys, and immune system. Hence, the development of highly sensitive and field-deployable detection strategies is of paramount importance. In this regard, SNAH-based sensing systems have been reported for multiple mycotoxin detection. For example, Lin et al. [168] constructed a DNA hydrogel incorporating the T-2 toxin aptamer and a copper-based metal–organic framework (Cu_3_(HHTP)_2_). Upon target binding, the hydrogel disintegrated, releasing MOF particles that catalyzed TMB color development for qualitative analysis, while their photothermal effect under laser irradiation enabled quantitative detection in grain samples. For ZEN detection, Sun et al. [169] developed a hyaluronic acid–DNA hybrid hydrogel sensor that integrated ZEN aptamers into the network and employed hybridization chain reaction amplification on Ce–Zr bimetallic MOFzymes. Binding-induced hydrogel degradation exposed the MOFzyme surface, catalyzing substrate oxidation and enabling ultrasensitive colorimetric detection in maize and soybeans. FB1 was detected using a magnetic bead-assisted entropy-driven catalytic cycle, where aptamer-functionalized DNA tetrahedra released catalytic DNA upon target recognition, triggering hydrogel cleavage and exposure of embedded Mn–Zr MOFzymes. This system achieved an ultralow detection limit of 0.38 pg/mL and demonstrated potential for multiplexed detection [170]. For aflatoxin B1 (AFB_1_), Li et al. reported an “on–off” fluorescence sensor using upconversion nanoparticles (CSUCNPs) and ZIF-8@Cu^2+^ composites, while Hao et al. [171] realized highly sensitive OTA detection in beer through aptamer-triggered RCA that generated fluorescent DNA hydrogels. Notably, SNAHs also support multiplexed detection. Aran et al. [172] constructed a dual-responsive hydrogel system capable of simultaneously detecting chloramphenicol (CAP) and aflatoxin M1 (AFM1), achieving visual sol–gel transition and fluorescence readouts in milk, thereby offering valuable insights into multi-analyte food safety analysis.

Beyond mycotoxins, SNAHs have been applied to the detection of algal toxins, foodborne pathogens, and illegal additives, underscoring their versatility and on-site applicability. For algal toxins, Wu et al. [173] designed a microcystin (MC)-LR responsive DNA hydrogel encapsulating Cu/Au/Pt trimetallic nanozymes. Target binding led to hydrogel disassembly and nanozyme release, catalyzing the TMB–H_2_O_2_ color reaction for sensitive colorimetric detection in water and fish samples. In pathogen detection, Mann et al. [174] reported an Escherichia coli-responsive DNAzyme hydrogel sensor triggered by the specific protein ECP1. Upon activation, DNAzyme-mediated hydrogel degradation released embedded AuNPs, generating colorimetric signals. Coupled with machine learning, this system enabled the accurate and automated identification of *E. coli* in complex matrices such as clinical urine, highlighting its potential in water monitoring and rapid diagnostics. Illegal additives represent another critical focus of food safety regulation. Wang et al. [175] developed a melamine (MEL) aptamer-based hybrid hydrogel, where MEL binding disrupted the hydrogel and released AuNPs, producing visual or spectrophotometric colorimetric signals. Integrated with microfluidic chips and a smartphone readout, this system enabled portable, quantitative detection of MEL in dairy and pet food. Similarly, Bian et al. [176] reported a ractopamine (RAC) aptamer-crosslinked hydrogel embedding Au@Pd bimetallic nanozymes. RAC binding induces hydrogel collapse and nanozyme release, significantly amplifying colorimetric responses for ultrasensitive detection in meat samples.

The applications of SNAHs in biosensors for food safety are shown in Table 3. Undoubtedly, SNAHs show significant potential for contaminant detection, despite facing notable challenges in complex food matrices. Their compatibility with portable platforms, such as microfluidic devices [177] and smartphone-based systems, supports their field-ready use in food safety supervision.

### 4.3. Disease Diagnosis

The core of biosensors in disease diagnosis lies in the highly precise monitoring of specific biomarkers [179]. Pathological processes, including the onset and progression of diseases, are frequently associated with characteristic alterations in biomolecules. These changes, which may involve the expression levels of specific proteins, metabolic small molecules, viral antigens, or the occurrence of structural abnormalities, provide a critical foundation for early diagnosis. Conventional hydrogel-based biosensors often encapsulate enzymes to achieve colorimetric sensing, as demonstrated in platforms for monitoring metabolites like lactic acid [180]. In contrast, SNAH-based biosensors relying on their specific recognition units can capture target molecules and leverage the environmentally responsive properties of hydrogels to convert biorecognition events into detectable signals, thereby enabling rapid and sensitive disease diagnosis [35,181].

In cancer diagnostics, alpha-fetoprotein (AFP) serves as an important serum biomarker for hepatocellular carcinoma, with significant clinical value [182]. Wang et al. [183] proposed a DNA hydrogel-based sensor, in which an AFP-specific aptamer was used as a crosslinker to construct a three-dimensional network. When AFP binds to the aptamer, the hydrogel structure disassembles and releases the pre-encapsulated IgG. The released IgG then forms a sandwich complex with SERS probes and magnetic beads. After magnetic separation, the decrease in SERS signal intensity in the supernatant allows for the quantitative detection of AFP. This strategy is not only applicable to liver cancer screening but can also be extended to other tumor biomarkers by replacing the aptamer with a corresponding one. For cancer therapy monitoring, Borum et al. [184] developed an injectable DNA hydrogel system for real-time monitoring of chemotherapeutic drugs. This system used a DNA-crosslinked hydrogel that has a high affinity for doxorubicin-methylene blue conjugates (MB-Dox). In the tumor microenvironment, the hydrogel is enzymatically degraded, releasing MB-Dox. The alteration in the aggregation state of MB-Dox resulting from this degradation leads to enhanced and red-shifted photoacoustic signals. By integrating multi-wavelength photoacoustic imaging, the system allows for dynamic monitoring of drug release. Beyond protein biomarkers, miRNAs have also emerged as crucial indicators for early cancer diagnosis. Si et al. [185] developed a sensor array based on multicomponent nucleic acid enzymes (MNAzymes), in which the recognition unit consists of DNAzymes (activated by specific miRNAs) embedded in a DNA hydrogel. When target miRNAs are present, the MNAzymes are activated to cleave the hydrogel crosslinkers, leading to hydrogel disintegration and the release of previously blocked SERS tags—this converts the signal from an “off” to an “on” state. This platform enables the quantitative detection of multiple cancer-related miRNAs in serum, achieving a detection limit as low as 0.11 nM and providing a promising approach for early cancer screening.

In infectious disease diagnosis, the rapid detection of viral nucleic acids is of paramount importance. To this end, functional nanomaterials such as graphene [186] and QDs have been integrated with specific molecular recognition elements to construct advanced biosensing platforms. For instance, Xu et al. [187] designed a QD–aptamer sensor including a QD fluorescent reporter gene. This system relies on the specific binding between the aptamer and the viral target, which induces volumetric changes in the hydrogel matrix. Subsequently, this physical transformation alters the fluorescence emission of the embedded QD reporters, enabling rapid, on-site monitoring of the virus. In a separate approach aimed at instrument-free detection, Wonhwi Na et al. [143] reported a microfluidic sensor that operates without external instrumentation. The system uses primers immobilized on microbeads and dumbbell-shaped DNA as recognition templates. Upon the presence of target viral RNA, a ligase-mediated reaction initiates RCA, producing long DNA strands that self-assemble into a macroscopic hydrogel. The formation of this hydrogel physically obstructs the microfluidic channels, providing a direct visual readout. This platform demonstrated the capability for simultaneous detection of multiple pathogens, including Ebola virus and MERS-CoV, within 15 min, highlighting its significant potential for rapid on-site screening. In the field of infection and metabolic monitoring, hydrogel sensors also show distinctive advantages [188]. Li et al. [189] designed a DNAzyme-responsive wireless sensor, in which the DNAzyme secreted by Staphylococcus aureus degrades the hydrogel, causing changes in electrical signals. By integrating near-field communication (NFC), wound infection monitoring can be conducted via smartphones. Additionally, Žuržul et al. [4] developed a potassium ion sensor based on G-quadruplex conformational transitions. The binding of K^+^ ions to the aptamer induces changes in hydrogel swelling; these changes are converted into optical signals via fiber interferometry, enabling the continuous monitoring of K^+^ in blood and providing valuable support for electrolyte management in intensive care. For in vivo monitoring, Li et al. [190] further designed an aptamer-based sensor suitable for physiological environments. Target binding induces conformational changes to regulate electrochemical signal output, while an agarose hydrogel coating effectively reduces biofouling and signal drift. This device enables the real-time monitoring of small molecules (such as antibiotics) in whole blood, tissues, and even live blood vessels, exhibiting excellent biocompatibility and clinical application potential.

The applications of SNAHs in biosensors for disease diagnosis are shown in Table 4. Research shows that peptide-functionalized DNA hydrogels are increasingly being explored in cell culture and tissue engineering, where they serve as intelligent coating materials with potential for integration into diagnostic platforms [191]. Furthermore, the combination of MIP technology with aptamers enhances the stability and selectivity of recognition units [192]. MIP-functionalized DNA hydrogels are expected to achieve more efficient target capture and signal transduction in complex biological samples, driving disease diagnostics toward higher sensitivity, greater multifunctionality, and real-time continuous monitoring.

## 5. Conclusions and Prospects

SNAHs are regarded as the core breakthrough of the next generation of intelligent biosensing units. Innovatively, this review proposes an integrated “recognition-decision–execution” framework that systematically maps diverse SNAH designs to specific stimuli and application scenarios. This framework provides a unified conceptual model for the rational design of intelligent sensing systems, thereby highlighting the unique conceptual advancement of this review over previous summaries. We systematically summarize the design principles of SNAHs based on molecular recognition modules, their stimuli-responsive mechanisms, and recent advances in applications spanning environmental monitoring, food safety, and disease diagnosis. From a construction perspective, different recognition elements exhibit distinct integration strategies. Specifically, aptamers and DNAzymes can serve as intrinsic structural components of hydrogels fabricated via nucleic acid self-assembly techniques like branched crosslinking or RCA. In contrast, other recognition elements are typically incorporated through chemical conjugation or physical encapsulation. Crucially, SNAHs are not merely passive, inert scaffolds. Leveraging the programmability of nucleic acids, they can actively function as integrated sensing elements, thereby unifying the scaffold and recognition functions—a distinctive advantage in biosensing. Overall, recognition modules function as the sensing triggering elements of SNAHs, while diverse physical, chemical, and biological responsive mechanisms provide pathways for signal transduction and output. The synergy between these two components establishes complete sensing circuits. Through flexible combinations of recognition units and responsive modes, researchers are able to design customized sensing platforms for specific targets, offering crucial technological support across environmental health, food safety, and biomedical diagnostics. Looking forward, SNAHs for biosensing applications still face key challenges as well as promising opportunities for development.

1. **Expansion of recognition targets and development of next-generation intelligent responsive modules.** Future advancements of SNAHs should prioritize the fundamental optimization of sequence stability, structural precision, and selective recognition before enabling more advanced smart sensing functions. Correspondingly, the next research phase should focus on enhancing AI- or machine learning-assisted nucleic acid sequence engineering to improve binding affinity, prediction accuracy, and structural robustness. Furthermore, the rational integration of non-natural functional groups (e.g., hydrophobic moieties, redox-active groups, photocrosslinkers, and metal-coordination sites, etc.) should be guided by well-documented mechanisms derived from existing SNAH systems, rather than being proposed as a generic strategy.
2. **Advancement of multiplexed detection and multimodal signal output strategies.** To address the growing demand for parallel, high-content analysis, future SNAH systems should focus on integrating multiple recognition modules with well-defined logic-gating circuits to achieve accurate multiplexed decision-making. This step represents the second tier of development, which is only feasible after resolving foundational issues related to stability and specificity. Regarding external stimuli, the systems reviewed in this work suggest several priority directions. Optical stimuli offer high spatiotemporal precision suitable for in vitro and wearable monitoring; magnetic fields provide deep-tissue penetration and remote actuation ideal for in vivo sensing; electrical stimulation is highly compatible with flexible electronics. Importantly, machine learning-guided optimization of hydrogel composition, porosity, and signal-transduction pathways could offer automated strategies for improving multiplex sensing fidelity.
3. **Promotion of theranostic integration, microfluidic convergence, and wearable applications.** Given their biocompatibility and programmability, SNAHs offer substantial advantages for future translational use. Beyond conventional drug delivery, hybrid organic–inorganic or multi-stimuli composite hydrogels (e.g., SNAH-MOF, SNAH-metal nanoparticle systems) could enable high-performance theranostic platforms with amplified sensing and therapeutic functions. A particularly important direction is the integration of SNAHs with microfluidic systems, flexible electronics, and wearable platforms. Microfluidics can provide precise sample manipulation and automated cascaded analysis, while flexible or epidermal electronics allow for continuous, on-body sensing with real-time feedback. Furthermore, embedding SNAHs in microneedle patches, strain-responsive hydrogel circuits, or self-powered sensing devices may enable next-generation personalized health monitoring with closed-loop intervention capability.
4. **Enhancement of matrix stability, device standardization, and pathways toward scalable industrial translation.** Prior to the translation of SNAH-based biosensors into practical or commercial applications, several foundational challenges must be effectively addressed. These include nonspecific adsorption, susceptibility to nucleases, mechanical fragility, and environmental interference—all of which directly affect device reproducibility and lifespan. Therefore, priority should be given to chemical modification (e.g., backbone stabilization, protective coatings, zwitterionic surfaces), mechanical reinforcement, and stabilization strategies validated in the current literature. To enable cross-laboratory reproducibility, standardized fabrication protocols, quality-control metrics, and benchmark testing systems should be established. Moreover, coupling SNAHs with low-cost mass-production techniques such as microfluidic extrusion, injection molding, or 3D printing represents an important pathway toward industrialization.

## Figures and Tables

**Table 1:** Recognition elements and their key characteristics in SNAH.

Recognition Element	Principle	Characteristics
Aptamer	Single-stranded nucleic acids that fold into specific 3D conformations to bind targets via non-covalent interactions.	High affinity and selectivity (nM–pM) Chemical stability Facile modification Prone to nuclease degradation.
DNAzymes	Catalytic DNA sequences capable of mediating substrate cleavage or redox reactions, typically dependent on specific metal ions.	Catalytic and signal-amplifying Metal-ion selective Functions as a responsive crosslinker Activity modulated by ions and environment
Antibody	Protein receptors recognizing antigens through epitope-paratope complementarity.	Excellent affinity and biological relevance High cost Low stability under extreme pH/temperature
Peptides	Short amino acid chains interacting with targets via specific motifs or secondary structures.	Biocompatible Easily synthesized and modified Lower affinity and selectivity (μM range)
MIPs	Synthetic polymers with template-shaped cavities formed during polymerization.	Resistant to harsh conditions Scalable fabrication Limited by incomplete template removal and heterogeneous sites

**Table 2:** Application of SNAHs in biosensors for environmental monitoring.

Analyte	Recognition Element	Response Mechanism	Sensor Strategy	LOD	Reference
Pb^2+^	DNAzyme	Cleavage of substrate strand induces hydrogel collapse, releasing nucleotide fragments.	Label-free pure DNA hydrogel biosensor	7.7 nM	[154]
Pb^2+^	DNAzyme	Enzyme-activated film degradation releases AuNPs for colorimetric readout.	DNAzyme–AuNP colorimetric sensor	2.6 nM	[156]
Pb^2+^	DNAzyme	Hydrogel mesh-size alteration modulates capillary flow velocity.	Capillary-based flow rate sensor	10 nM	[38]
Hg^2+^	T-rich DNA sequence	Specific T–Hg^2+^–T coordination complex formation, generating a fluorescent signal.	DNA-functionalized fluorescent hydrogel sensor	0.05 nM	[153]
UO_2_^2+^	DNAzyme	Film disintegration via enzymatic cleavage releases AuNPs for colorimetric detection.	DNAzyme-mediated AuNP release-based colorimetric sensor	10.3 nM	[156]
UO_2_^2+^	DNAzyme	Hydrogel breakdown releases Raman reporters, which are captured and enhanced by Ag-NPs@PAN membrane for SERS.	SERS-enhanced flexible hydrogel sensor	0.838 pM	[155]
Ln^3+^	DNAzyme	Hydrogel collapse releases AuNPs, resulting in a color change.	Colorimetric biosensor	20 nM	[161]
Malathion	Aptamer	Competitive binding disrupts hydrogel network, releasing AuNPs (colorimetric) and reducing Fc-labeled aptamer (electrochemical).	Dual-mode colorimetric/electrochemical microfluidic chip sensor	56 nM	[157]

**Table 3:** Application of SNAHs in biosensors for food safety.

Analyte	Recognition Element	Response Mechanism	Sensor Strategy	LOD	Reference
T-2	Aptamer	Target-induced release of the nanozyme Cu_3_(HHTP)_2_ from a DNA hydrogel catalyzes TMB oxidation, enabling dual colorimetric and photothermal readout	Dual-modal colorimetric and photothermal sensor	1.67 ng/mL	[168]
ZEN	Aptamer	ZEN-aptamer binding triggers hydrogel disintegration, exposing encapsulated MOFzyme that catalyzes substrate reaction	MOFzyme-based TMB colorimetric sensor	0.8 pg/mL	[169]
FB_1_	Aptamer	FB_1_ binding releases DNA strands to initiate a displacement reaction, leading to hydrogel dissolution and MOFzyme release	MOFzyme-based TMB colorimetric sensor	0.38 pg/mL	[170]
AFB_1_	Aptamer	AFB_1_ binding enhances electrostatic repulsion between probes, disrupting FRET effect and turning on CSUCNPs fluorescence	Turn-on fluorescent biosensor	0.08 μg/kg	[178]
OTA	Aptamer	OTA binding releases primers to initiate RCA, generating long fluorescent DNA chains for signal amplification	Fluorescence signal-amplified biosensor	0.01 ng/mL	[171]
MC-LR	Aptamer	Hydrogel dissociation releases encapsulated Cu/Au/Pt trimetallic nanoparticles (TNs)	Colorimetric sensor	3.0 ng/L	[173]
*E. coli*	DNAzyme	DNAzyme cleavage degrades hydrogel network, releasing AuNPs for colorimetric response	DNAzyme-mediated AuNP release-based colorimetric sensor	10^1^ CFU mL^−1^	[174]
RAC	Aptamer	Hydrogel collapse releases Au@Pd nanozymes that catalyze the H_2_O_2_-TMB reaction.	Ultrasensitive optical colorimetric sensor	7.39 ng/L	[176]
MEL	Aptamer	Hydrogel dissociation releases entrapped AuNPs.	Colorimetric biosensor	37 nM	[175]

**Table 4:** Application of SNAHs in biosensors for disease diagnosis.

Analyte	Recognition Element	Response Mechanism	Sensor Strategy	Reference
*S. aureus* DNase	DNA hydrogel	Enzymatic degradation of the hydrogel by DNase induces a change in dielectric properties	NFC module integrated with a smartphone for wireless readout	[189]
K^+^	Aptamer	Target binding induces a concentration-dependent de-swelling of the aptamer-functionalized hydrogel	aptamer-hydrogel-based interferometric fiber sensor	[4]
MB-Dox	DNA-Crosslinked Hydrogel	Hydrogel degradation in response to endogenous nuclease activity releases MB-Dox, enhancing the photoacoustic signal	Enzyme-responsive disintegration of a DNA hydrogel matrix for turn-on photoacoustic imaging	[184]
Kanamycin	Aptamer	A hydrogel-coated aptamer sensor generates an electrochemical signal upon binding while providing antifouling capability	Electrochemical sensor based on a DNA aptamer with a protective hydrogel layer	[190]
AFB	Aptamer	Dissociation of the hydrogel network releases pre-embedded IgG, facilitating the formation of a sandwich complex with SERS probes and functionalized magnetic beads	Aptamer-responsive DNA hydrogel-based SERS biosensor	[183]
miRNA	MNAzymes	MNAzyme cleaves crosslinking substrates within the DNA hydrogel, leading to hydrogel dissolution and activation of the SERS signal	MNAzyme-responsive DNA hydrogel-based SERS biosensor	[185]
Virus	DNA template	Assembly of long DNA strands into a hydrogel network blocks the microfluidic pathways between beads	Microfluidic chip-integrated DNA hydrogel sensor	[143]
Glucose	Aptamer	Target binding triggers the release of pre-encapsulated gold nanoparticles (AuNPs) from the hydrogel	Colorimetric biosensor based on an aptamer-functionalized hydrogel	[193]

## Abbreviations

3D	three-dimensional
AFM1	aflatoxin M1
AFP	alpha-fetoprotein
AFs	aflatoxins
AgNCs	silver nanoclusters
AMPs	antimicrobial peptides
APC	antigen-presenting cell
APS	ammonium persulfate
ATP	adenosine triphosphate
AuNPs	gold nanoparticles
AuNRs	gold nanorods
CAP	chloramphenicol
cDNA	complementary DNA
COR	coralyne
CTLA-4	cytotoxic T lymphocyte antigen 4
DGT	diffusive gradients
DOX	doxorubicin
EDC/NHS	1-Ethyl-3-(3-dimethylaminopropyl)carbodiimide/N-Hydroxysuccinimide
ELISA	enzyme-linked immunosorbent assay
EpCAM	epithelial cell adhesion molecule
FB1	fumonisin B1
FLD-MS	fluorescence detection–mass spectrometry
GC	gas chromatography
HPLC	high-performance liquid chromatography
HRCA	hyperbranched rolling circle amplification
HRP	horseradish peroxidase
ITO	Indium-tin oxide
LC	liquid chromatography
LCST	lower critical solution temperature
LOD	limit of detection
LPS	lipopolysaccharides
MAF	malathion aptamer
MBAAm	methylene bis(acrylamide)
MB-Dox	doxorubicin-methylene blue conjugates
MC-LR	Microcystin-LR
MEL	melamine
microRNA	miRNA
MIPs	molecular imprinted polymers
MNAzymes	multicomponent nucleic acid enzymes
MNPs	magnetic nanoparticles
MOFs	metal–organic frameworks
MUC1	mucin 1 protein
NFC	near-field communication
NIR	near-infrared
nM	nanomolar
OTA	ochratoxin A
PCR	polymerase chain reaction
PD-1	cell death protein 1
pM	picomolar
pNIPAM	poly(N-isopropylacrylamide)
QD	quantum dots
RAC	ractopamine
RCA	rolling circle amplification
RhB	rhodamine B
ROS	reactive oxygen species
SELEX	Systematic Evolution of Ligands by Exponential Enrichment
SEM	scanning electron microscopy
SERS	surface-enhanced Raman scattering
SNAHs	smart nucleic acid hydrogels
SPR	surface plasmon resonance
ssDNA	single-stranded DNA
T cell	T lymphocytes
TBO	toluidine blue O
TEMED	N,N,N′,N′-tetramethylethylenediamine
tFNAs	tetrahedral framework nucleic acids
TGMS	Triglycerol monostearate
TMB	3,3′,5,5′-tetramethylbenzidine
TNF-α	tumor necrosis factor-α
UO_2_^2+^	uranyl ion
V.P.	Vibrio parahaemolyticus
ZEN	zearalenone
μM	micromolar
